# Évaluation de la prévention des infections associées aux soins dans des cabinets dentaires à Rabat

**DOI:** 10.11604/pamj.2023.45.106.35297

**Published:** 2023-06-26

**Authors:** Marrouk Wiam, El Basraoui Ghita, Haj khalaf Lauzan, Toure Babacar

**Affiliations:** 1 International University of Rabat, College of Health Sciences, International Faculty of Dental Medicine, BioMed Unit, Technopolis Parc, Rocade of Rabat-Salé, Sala-Al Jadida, 11100, Morocco

**Keywords:** Soins dentaires, infections croisées, étude transversale, dentistes, asepsie, stérilisation, Dental care, cross infections, cross-sectional study, dentists, asepsis, sterilization

## Abstract

**Introduction:**

en dentisterie, plusieurs traitements sont invasifs et peuvent être responsables d'apparition des infections. L'objectif de cette étude était d'évaluer la prévention des infections associés aux soins au cabinet dentaire de Rabat.

**Méthodes:**

une enquête sur la prévention des infections associées aux soins dans des cabinets de Rabat a été menée auprès de 324 praticiens. Un questionnaire auto-administré a été utilisé afin d'évaluer leurs formations, l'hygiène, la protection, l'organisation des locaux, l'asepsie, antisepsie et l'utilisation des dispositifs médicaux. Les données ont été collectées sur JAMOVI version 1.8.4, les tests χ^2^ et Fisher ont été utilisés pour comparer les variables, le niveau significatif est P<0.05.

**Résultats:**

seuls 80 ont participé à l'étude. Tous les dentistes disposent de moyens de protection, cependant 68 (85%) n'ont pas la fiche de conduite à tenir en cas d'accident d'exposition au sang. Quarante-un praticiens (62,1%) ont des assistants diplômés, 36,4% ont une équipe vaccinée contre l'hépatite B et 61,3% des praticiens et des assistants ont été formés pour le traitement des dispositifs médicaux réutilisables. Soixante-seize cabinets ont des salles d'examen isolées, 41 disposent d'un stérilisateur à vapeur ainsi qu'une zone de tri disposant d'un collecteur d'objet piquant coupant et tranchant. Cependant 35,1% disposent d'un contrat d'enlèvement des déchets d'activités des soins à risque infectieux.

**Conclusion:**

les résultats de cette étude montrent que la plupart des praticiens exerçant à Rabat respectent la majorité des normes d'asepsie et d'hygiène de la dentisterie. Cependant des efforts doivent être renforcés en ce qui concerne la vaccination du personnel.

## Abstract (en)

**Introduction:**

in dentistry, many treatments are invasive and can lead to infections. The purpose of this study was to evaluate the prevention of healthcare-associated infections in the dental practice in Rabat.

**Methods:**

a survey on the prevention of healthcare-associated infections in Rabat dental practices was conducted among 324 practitioners. A self-administered questionnaire was used to assess their training, hygiene, protection, organization of premises, asepsis, antisepsis and use of medical devices. Data were collected using JAMOVI version 1.8.4, the χ^2^ and Fisher tests were used to compare the variables, the significance level was set at P<0.05.

**Results:**

only 80 subjects took part in the study. All the dentists had protective equipment, but 68 (85%) did not have instructions on what to do in case of accidental exposure to blood; 41 (62.1%) operators had qualified assistants, 36.4% had a team vaccinated against hepatitis B and 61.3% of operators and assistants had been trained to handle reusable medical devices. Seventy-six studies had isolated examination rooms, 41 had a steam steriliser and a sorting area with sharps collector. However, 35.1% had a contract for the collection of waste generated by health-care activities, posing a significant risk of infection.

**Conclusion:**

the results of this study show that most practitioners in Rabat comply with the majority of the aseptic and hygienic standards in dentistry. However, more needs to be done to ensure that staff are vaccinated.

## Introduction

La pratique dentaire est généralement accompagnée par le risque de contamination à la suite de la projection des aérosols et des contacts manuels des intervenants avec l'environnement, la raison pour laquelle, il est nécessaire d'assurer une protection maximale aussi bien par les praticiens que par leurs assistants [1] et d'entretenir les équipements et les surfaces [2] afin de protéger leur propre santé et la santé de leurs patients. Plusieurs conditions sont susceptibles de compromettre la qualité des soins et des actes dispensés ainsi que la sécurité des patients notamment l'organisation des locaux [3] et le traitement des dispositifs médicaux qui sont classés en trois catégories: critiques de haut risque de transmission, pénètrent dans les tissus ou cavités stériles ou dans le système vasculaire: semi-critiques avec un risque médian de transmission qui entrent en contact avec la muqueuse buccale et la salive et dispositifs médicaux non critiques de faible risque de transmission qui n'ont pas de contact direct avec le patient, cela signifie que le niveau de traitement des dispositifs médicaux est déterminé prioritairement en fonction du risque infectieux potentiel lié à l'indication de ceux-ci [4].

Les infections associées aux soins dentaires issues des pratiques dentaires peuvent être à l'origine de plusieurs crises sanitaires sujets de scandales de santé publique, la raison pour laquelle certains guides ont été rédigés pour aider le chirurgien-dentiste à prévenir le risque de transmission d'agents pathogènes permettant aux professionnels et aux structures de soins dentaires d'analyser leur pratique et de définir des actions d'amélioration [5]. Depuis la disponibilité de ses guides aucune étude n'a analysé le niveau du risque des infections associées aux soins dans les cabinets privés au Maroc. Dans ce contexte cette étude a été faite dont l'objectif était d'évaluer les mesures de prévention mises en œuvre par les médecins dentistes privés de Rabat pour lutter contre les infections associées aux soins.

## Méthodes

**Cadre de l'étude:** il s'agit d'une étude descriptive transversale menée entre avril et juin 2021 auprès des médecins dentistes inscrits au niveau de l'ordre régional et exerçant à la ville de Rabat.

**Contexte organisationnel:** la présente enquête était exhaustive en ciblant tous les médecins dentistes. Cette étude a été approuvée par le conseil de l'Ordre des Médecins Dentistes qui a remis la liste de tous les dentistes de la ville de Rabat. Cette liste comporte les informations suivantes: nom et prénom, adresse du cabinet et adresse électronique du praticien. L'enquête a été réalisée par un questionnaire auto administré inspiré de la grille de l'ADF (Association Dentaire Française) sur l'évaluation technique des cabinets dentaires pour la prévention des infections associées aux soins.

Le questionnaire a été rédigé sur Google Forms et comporte 2 parties: la première partie relative aux données socio démographiques des praticiens et la deuxième partie évalue les mesures de prévention mises en œuvre pour lutter contre les infections associées aux soins. Cette deuxième partie a été subdivisée en 4 rubriques. Cette enquête, était strictement anonyme, aucune question ne demandait aux participants d'informations personnelles, susceptibles de permettre de les identifier à posteriori. Le questionnaire était introduit par un texte explicatif et les questions étaient fermées, à propositions multiples ou ouvertes. Il a été envoyé par courrier électronique à toutes les adresses emails valides fournies par le conseil régional nord.

**Participants:** pour être retenus dans l'étude, il fallait: être docteur en médecine dentaire et régulièrement inscrit à l'Ordre National des Médecins Dentistes du Maroc (ONMDM): exercer la médecine dentaire à titre privé à la ville de Rabat: être disponible et accepter de participer à l'étude et disposer une adresse électronique valide.

**Variables:** les variables socio démographiques étaient l'âge, le sexe, l'ancienneté d'exercice et le type de cabinet en groupe ou individuel; tandis que les variables des mesures de la prévention contre les infections associées aux soins étaient la formation, hygiène et protection du personnel, locaux, asepsie et utilisation des dispositifs médicaux.

**Sources de données:** cette étude a été approuvée par le conseil de l'Ordre des Médecins Dentistes qui a remis la liste de tous les dentistes de la ville de Rabat. Selon la liste fournie par le conseil régional nord de l'ordre des médecins dentistes, il existe 471 cabinets privés sur la ville de Rabat en 2021 [6]. L'enregistrement des données a été effectué sur la feuille de calcul fournie automatiquement après les réponses (Sheets sur Google drive). Puis ses informations recueillies ont été codées en chiffres pour faciliter le transfert et le traitement dans le logiciel JAMOVI.

**Biais:** un rappel régulier par mail a été fait chaque 15 jours. En parallèle, le questionnaire a été aussi envoyé sur les groupes WhatsApp et Facebook des médecins dentistes de Rabat.

**Taille de l'étude:** il existe 471 cabinets privés sur la ville de Rabat en 2021 [6], en revanche uniquement 324 adresses électroniques des médecins dentistes qui ont pu être obtenues par le conseil de l'Ordre des Médecins Dentistes.

**Comment les variables ont été exprimées:** les données quantitatives: l'âge et l'ancienneté d'exercice ont été exprimées en moyenne et écart type et les données qualitatives en nombre et pourcentage.

**Méthodes statistiques:** le logiciel JAMOVI version 1.8.4 a permis d'analyser ses données et le test χ^2^; et Fisher ont été utilisés pour comparer les variables entre elles. Le niveau de signification a été fixé à P<0.05.

## Résultats

**Figure 1:**
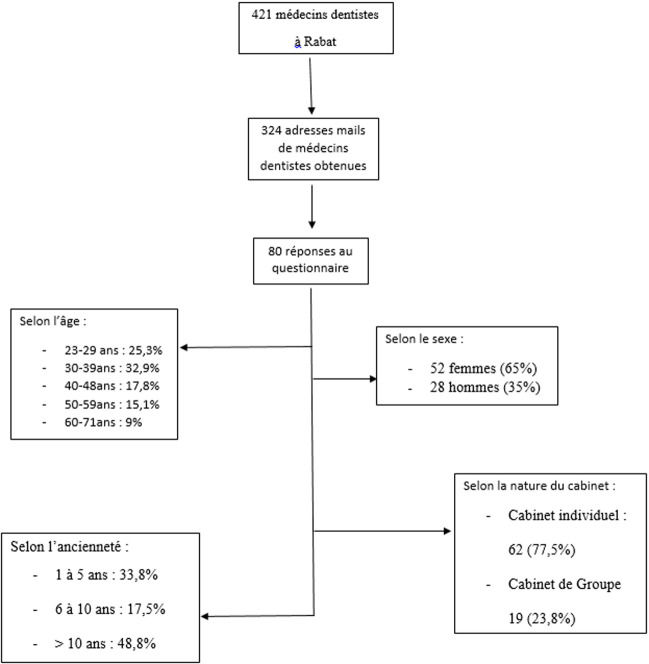
caractéristiques sociodémographiques de la population étudiée

**Caractéristiques sociodémographiques de la population étudiée:** l'enquête a intéressée 324 médecins dentistes inscrits à l'ordre et exerçant dans la ville de Rabat, cependant 80 ont répondu au questionnaire présentant un taux de participation de 24,69%. Les variables considérées ont été le sexe, l'âge, l'ancienneté dans l'exercice de la profession et le type d'activité (individuel ou en groupe). Sur les 80 participants 52 sont de sexe féminin soit 65% et 28 de sexe masculin soit 35%. Les praticiens ayant une expérience au-delà de dix ans représentent 48,8%. Les cabinets individuels étaient les plus représentés 62 soit 77,5, les femmes avaient plus tendances à travailler dans les cabinets de groupe (26,92%) que celle des hommes (14,26%). L'âge moyen des participants était de 39,6±12,2 ans avec le plus jeune âgé de 23 ans et le plus âgé de 71 ans. La tranche d'âge la plus représentée est celle de 30-39ans (32,9) (Figure 1).

### Les mesures de prévention des infections associées aux soins

***Formation du personnel:*** les assistants qualifiés et diplômés représentées 61,2% tandis que les 25 restants soit 37,9% n'ont pas de qualifications mais ils ont été formés au sein des cabinets. En ce qui concerne le traitement des dispositifs médicaux réutilisables (DMR), une formation spécifique réactulaisée a été suivie que par 46,3% du personnel asssitant. En revanchecette formation a été suivie par 61,3% des médecins avec une prédominace féminine de65,38%. En comparant le sexe chez les hommes la formation était plus élevée chez les praticiens ayant une expérience au-delà de 10ans (10 contre 5) contrairement chez les femmes le taux de formaion était plus élevé chez les plus jeunes (19 contre 15) mais la différence n'était pas significatif (P=0,301).

***Protection de l'équipe soignante:*** concernant les masques ils sont portés par l'ensemble des praticiens. Pour les dispositifs de protection oculaires et faciles 42,5% les utilisent lors des soins sans aérosol, 80% dans les soins aérosolisants et non-souillant et 83,8% en cas de soin aérosolisant souillant. Sur les 80, 68 soit 85% ont déclaré ne pas avoir la fiche ‘Conduite à tenir en cas d'accident d'exposition au sang ‘AES’’ dans leurs cabinets. La disponibilité de cette fiche détaillant la conduite à tenir face à un accident d'exposition au sang a été notée seulement chez 12 praticiens dont 2 hommes et 10 femmes. Par ailleurs la fiche complète avec le numéro du médecin urgentiste à contacter n'était présente que chez 6 praticiens. L'absence de cette fiche était plus marquée dans les cabinets individuels (91,93%) que dans les cabinets de groupe (61,11%) avec P=0,004.

Par rapport à la vaccination, seuls 31 praticiens soient 39,2% ont déclaré être informés du statut vaccinal de leurs personnels versus 60,8% qui l'ignorent c'est-à-dire n'ont aucune connaissance de la situation vaccinale de leurs assistants. En ce qui concerne la vaccination contre l'hépatite B, dans la majorité des cabinets (63,6%) l'équipe soignante n'était pas vaccinée contre l'hépatite lors que 36,4% présentant le tiers étaient à jour de sa vaccination contre le virus de l'hépatite B.

***Organisation des locaux:*** soixante-six praticiens soit 83,5% déclarent avoir une salle de soin contenant un lavabo à commande non manuelle, 69 soit 87,3% ont des distributeurs de solution hydroalcoolique et de savon liquide et 58 soit 73,4% ont des distributeurs d'essuie-mains à usage unique. Soixante-huit soit 86,1% déclarent avoir un local organisé pour que le sale (dispositifs médicaux souillé) n'entre pas en contact avec le propre (dispositifs médicaux contrôlés, emballés et stérilisés) contre 11 soit 13,9% ne disposent pas de local dédié au traitement des dispositifs médicaux. 85% ont répondu avoir des équipements faciles à entretenir type fauteuil lisse sans coutures, un système d'aspiration démontable et des commandes à pédales contre 15% qui n'ont disposent pas.

***Asepsie, antisepsie et acte dentaire:*** pour le maintien des équipements 51,2% des participants réalisent une purge des équipements en début de séance soit avant la 1^re^ utilisation de l'unit, 39 soit 48,8% la réalise après chaque patient tandis que 16 soit 20% ne réalisent pas de purge d'équipement. Un nettoyage des surfaces de proximité de l'unité avec un produit détergent ou désinfectent est assuré entre deux patients par 75 praticiens soit 93,8% dont la majorité sont des dentistes femmes 98,5%. Pour la désinfection de l'opercule de la cartouche d'anesthésie 37,5% des praticiens la réalise avant son utilisation dont 43,9% sont des dentistes jeunes. Pour les produits antiseptiques 96,3% respectent la dilution, le temps et la fréquence de renouvellement préconisés par le fabricant. En plus 43,6% des praticiens inscrivent les dates d'ouverture des produits antiseptiques et sur les flacons d'emballages. Pour les instruments détachables type turbine, contre angle, pièces à main ou à ultrasons, 65% les retirent après chaque soin pour être traités Le corps de la seringue air/eau est nettoyé et désinfecté extérieurement entre chaque patient pour 91,1% des dentistes. Un contrôle régulier de l'efficacité de nettoyage de leurs laveur-désinfecteurs est assuré par 50,6% des praticiens et 74,4% vérifient l'efficacité du nettoyage et du séchage de chaque dispositif médical avant le conditionnement en vue de la prise en charge à l'autoclave (Tableau 1).

**Tableau 1:** attitude et pratique de prévention

	Réalisé		Non réalisé
Réalisation d'une purge des équipements	**Avant**	**Après**	20%
	51,2%	48,8%	
Nettoyage des surfaces à proximité de l'unit entre 2 patients	93,8%		6,2%
	**Femme**	**Homme**	
	98,57%	85,71%	
Désinfection de l'opercule de la cartouche d'anesthésie	62,5%		37,5%
Respect des recommandations du fabricant	96,3%		3,7%
Inscription de la date d'ouverture sur les antiseptiques et autres flacons	43,6%		56,4%
Retrait des instruments détachables après chaque soin pour traitement séparé	65%		35%
Nettoyage et désinfection du corps de la seringue air/eau extérieurement entre patients	91,1%		8,9%
Contrôle régulier de l'efficacité de nettoyage du laveur-désinfecteur	50,6%		49,4%
Vérification de l'efficacité du nettoyage et séchage avant conditionnement	74,4%		25,6%
Immersion des dispositifs médicaux réutilisables	97,5%		2,5%
Contrôle de l'action des ultrasons	50,6%		49,4%

***Utilisation des dispositifs médicaux:*** soixante-dix-sept soit 97,5% ont déclaré faire une immersion de tous les dispositifs utilisés en bouche dès la fin de leur utilisation dans un bac d'une taille suffisante contenant une solution détergente/désinfectante exempte d'aldéhydes (Tableau 1). Quarante soit 50,6% ont un dispositif de traitement de l'eau et 39 soit 49,9% ne le possèdent pas dans leurs cabinets. Pour 50% des praticiens, l'efficacité de l'action des ultrasons est régulièrement contrôlée (Tableau 1). Pour 53 soit 66,3% contrôlent le document traçant tous les événements de l'autoclave. Sur 71 dentistes, 41 praticiens ont un stérilisateur à vapeur d'eau qui a fait l'objet d'une qualification opérationnelle sur site de son procédé de stérilisation. La température et la durée du plateau de stérilisation vérifiées par 51 dentistes soit 68,8%, le virage correct de l'intégrateur physico chimique contrôlé par 35,1% alors que le virage de tous les indicateurs de passage figurant sur les sachets n'est vérifié que par 54,1%. L'absence d'humidité des sachets et intégrité des emballages est vérifiée par 78,4% des dentistes Cependant le test Hélix (de pénétration de vapeur d'eau) est contrôlé par 14 médecins soit 18,9% et le test Bowie-Dick est vérifié par 2 dentistes soit 2,8%. La date de stérilisation est mentionnée sur l'étiquette des sachets stérilisés par 72,3% des dentistes, le numéro du cycle de stérilisation est inscrit sur 40,4% des sachets stérilisés et la date limite d'utilisation est mentionnée par 27,7%.

## Discussion

**Caractéristiques de la population étudiée:** l'étude transversale a été réalisée auprès de 324 médecins dentistes inscrits au niveau du conseil d'ordre régional nord et exerçant à la ville de Rabat et cela après plusieurs rappels qui ont été fait sur une période de deux mois pour avoir seulement 80 réponses représentant un taux de participation de 24,69% tout en notant un faible nombre de réponses pour certaines questions notamment les questions ouvertes. Une participation marquée des femmes a été notée 65% ce qui confirme les constats observés dans la médecine dentaire lié à la féminisation de ce milieu professionnel. Cette féminisation peut être justifiée par une augmentation globale du nombre des praticiens et non pas la diminution de leurs homologues masculins [7]. Une participation moins marquée des dentistes âgés de moins de 30 ans qui représentent uniquement le ⅕ de l'échantillon peut être expliquée d'une part par le coût du cabinet privé qui nécessite des moyens qui ne sont pas toujours à la portée des jeunes praticiens et d'autres part, par le fait que certains jeunes diplômés préfèrent acquérir une certaine expérience avant de s'installer [8]. L'enquête montre également que les chirurgiens-dentistes préfèrent la pratique individuelle à la pratique en groupe soit pour conserver l'autonomie ou bien pour d'autres raisons.

**Formation du personnel:** bien que l'objectif principal de l'étude est l'évaluation de la prévention des infections associées aux soins au cabinet dentaire, le contrôle de la formation du médecin et de son équipe soignante est tout aussi importante car ils participent à la prévention des différents risques associés à la pratique dentaire et l'amélioration de la qualité des soins. Cette enquête a montré que 61% des praticiens à Rabat déclarent avoir des assistants diplômés et qualifiés cependant dans une étude des connaissances et pratiques en hygiène hospitalière réalisée au CHU de Cocody d'Abidjan, montre que 72,73% des assistants dentaires pratiqués des gestes à hauts risque de contamination croisée [9].

**Protection de l'équipe soignante:** lors des soins dentaires certaines maladies peuvent être transmises du patient à l'équipe médicale et de l'équipe médicale au patient, la raison pour laquelle une protection est nécessaire. L'étude a montré que la totalité des praticiens disposent de masque et de protection faciale. Alors qu'une étude réalisée auprès des étudiants de la faculté de chirurgie dentaire de Lille en 2016 a montré que même si 92% des étudiants portaient des lunettes de protection lors des traitements, il s'agissait pour la majorité d'entre eux de leurs lunettes de vue classique, seulement un étudiant sur cinq qui nettoyait ses lunettes entre chaque patient et aucun personnel de stérilisation ne portait de protection oculaire lors des gestes nécessitant leur port [10]. Selon une étude du CHU de Cocody d'Abidjan moins du ⅓ de l'ensemble des personnels porte des lunettes de protection et d'après une étude effectuée en Algérie seulement 4,6% des praticiens disposent des équipements de protection individuel [9]. La fiche «conduite à tenir face aux accidents d'exposition au sang ‘AES’» n'est pas présente dans plus des ¾ des cabinets dentaires. Bien que la majorité des praticiens connaissent la conduite à tenir immédiate face aux ‘AES’, il est important qu'ils sachent les démarches à entreprendre avec le médecin urgentiste. Le statut vaccinal de chaque personnel travaillant dans la santé doit être actualisé et conforme aux normes [11,12]. Cette enquête a montré que les ⅔ (63,3%) de l'équipe soignante n'est pas vaccinée contre l'hépatite B, même si le risque de transmission de l'hépatite B à partir d'un patient infecté est de 2 à 40% avec une estimation moyenne de 30% (1 chance sur 3) en l'absence de prophylaxie vaccinale. Toutefois, depuis janvier 1991, date à laquelle la vaccination contre l'hépatite B a été rendue obligatoire en France, le risque de contamination est en nette régression. Selon une enquête menée entre 1984 et 1996: 7% des 142 dentistes avaient eu l'hépatite B en 1984 alors qu'en 1996, uniquement 3,7% avaient eu ce type d'hépatite. 80,2% des médecins dentistes consultés sont vaccinés en 1996 contre 20,4% en 1984 [13]. Une autre étude menée à Berlin en 1977 sur 215 dentistes et 108 assistants 7% des praticiens et 1% des assistants avaient eu de l'hépatite. Seulement 74% des dentistes et 63% des assistants ont déclaré être vaccinés [13].

**Organisation des locaux:** l'installation des locaux doit permettre une hygiène optimale qui fait partie des précautions standard permettant à elle seule de diminuer de façon très importante les contaminations croisées, la raison pour laquelle le local doit être organisé en trois zones: zone administrative (bureau), zone potentiellement contaminée (zone de traitement de matériel) et zone dite protégée (zone d'examen et de soins) [13], ainsi qu'il faut prévoir une bonne ventilation du local en raison de l'utilisation fréquente des solutions de produits désinfectants, de bac à ultrasons et d'autoclaves qui augmente la température ambiante [14]. Selon cette étude la majorité des praticiens de Rabat ont déclaré avoir des salles de soins individualisées et ¾ d'entre eux ont des équipements faciles à entretenir. Dans une étude réalisée au sein du service d'odontologie du CHRU de Lille en 2016 pour évaluer la gestion du risque infectieux, le service possédait bien un local spécifique de traitement des dispositifs médicaux dont l'objectif était de permettre une centralisation du processus de stérilisation des dispositifs médicaux utilisés [10].

**Asepsie, antisepsie et acte dentaire:** plusieurs études ont montré que les concentrations bactériennes retrouvées dans les circuits d'eau sont supérieures à celles du réseau de distribution d'eau potable dont elle provient, de ce fait il est nécessaire de diminuer la concentration microbienne de l'eau des circuits pour éviter la survenue des infections chez les patients ou le personnel du cabinet, cela est possible par la réalisation de purge permettant de réduire transitoirement la concentration microbienne par prolifération des micro-organismes [15]. Dans cette étude uniquement 51,2% réalisent une purge avant tout soin et 48,8% la réalisent après chaque patient, malgré qu'il ait été démontré que les purges de l'unit doivent être effectuées en début de journée pendant 5 minutes avant de connecter les portes instruments rotatifs, entre chaque patient d'une durée de 20 à 30 secondes lorsque les instruments dynamiques souillés sont encore mis en place et enfin de vacation pendant 20 secondes [16]. L'opercule de la cartouche d'anesthésie peut être à l'origine d'une contamination car il peut être infecté par les germes de l'environnement lors du stockage, lors des manipulations mais aussi par les aérosols présents dans les cabinets, alors que plus que la moitié des participants pratique l'anesthésie sans désinfection préalable [17]. Selon les recommandations du Ministère de la santé ‘DGS’ en 2006, toutes les portes instruments rotatifs (PIR) doivent être stérilisées (les pièces à main, contre angles et turbines). Pour cela ils doivent être débranchés pour assurer la désinfection des raccords avec les produits détergents désinfectant en même temps que l'unit pour limiter les contaminations croisées [16]. Dans cette présente enquête, 65% des dentistes désinfectent les PIR après chaque soin.

**Utilisation des dispositifs médicaux:** selon l'agence régionale de santé, il est obligatoire de souscrire un contrat d'entretien pour les dispositifs complexe (autoclave, laveur désinfecteur thermique et appareil à ultrasons) et de tenir un cahier de maintenance [17,18]. Dans cette étude la souscription à ce contrat a été faite par la moitié des dentistes de Rabat et des évènements du contrôle de l'autoclave qui permettent de prévenir toute panne ou défaillance influençant la qualité de stérilisation et la sécurité des utilisateurs [19,20] n'ont pas été retracé. Selon cette présente étude plus de la moitié des dentistes participants ont un stérilisateur à vapeur d'eau qui fait l'objet d'une qualification opérationnelle qui a été effectuée à partir de 3 cycles pour s'assurer de la reproductibilité des résultats conformément à la norme NF EN ISO 17665-1 [21] ce qui le qualifie d'un appareil de qualité.

## Conclusion

Les infections associées au soin sont un véritable problème de société que les pouvoirs publics tentent de résoudre depuis de nombreuses années. Elles peuvent être prévenues dans les cabinets dentaires si les normes et les protocoles de protection, d'hygiène et d'asepsie sont bien établis par l'équipe soignante. Selon cette étude, les chirurgiens-dentistes inscrits à l'ordre et exerçant en cabinet privé dans la ville de Rabat ayant accepté de participer à l'étude, respectent en partie les recommandations essentielles d'hygiène et d'asepsie. Cependant des améliorations peuvent être apportées surtout en termes de formation du personnel afin de garantir une meilleure ergonomie en matière de prévention des infections associées aux soins. Une enquête s'étendant à l'ensemble des praticiens marocains est nécessaire afin d'évaluer le niveau de la prévention des infections associées au soin au niveau du royaume.

### Etat des connaissances sur le sujet

- *Les infections associées aux soins dentaires présentent un véritable problème de santé publique, la raison pour laquelle plusieurs recommandations doivent être respectées pour prévenir leurs transmissions, protéger la santé des patients et de l'équipe soignante et garantir des soins de qualité*.

### Contribution de notre étude à la connaissance

- *Cette enquête permet d'évaluer le niveau de prévention des infections associées aux soins dans les cabinets privés de Rabat en 2021;*
- *L'étude permet de suggérer un certain nombre de recommandations pour renforcer le statut vaccinal du personnel et améliorer la qualité de la formation en matière d'hygiène et d'asepsie*.

## Remerciements

Les auteurs remercient le doyen du collège de santé de l'Université Internationale de Rabat Monsieur Benjouad Abdelaziz pour son aide.
